# Methylated nucleosides in tRNA and tRNA methyltransferases

**DOI:** 10.3389/fgene.2014.00144

**Published:** 2014-05-23

**Authors:** Hiroyuki Hori

**Affiliations:** Department of Materials Science and Biotechnology, Applied Chemistry, Graduate School of Science and Engineering, Ehime UniversityMatsuyama, Japan

**Keywords:** RNA modification, RNA methylation, RNA maturation

## Abstract

To date, more than 90 modified nucleosides have been found in tRNA and the biosynthetic pathways of the majority of tRNA modifications include a methylation step(s). Recent studies of the biosynthetic pathways have demonstrated that the availability of methyl group donors for the methylation in tRNA is important for correct and efficient protein synthesis. In this review, I focus on the methylated nucleosides and tRNA methyltransferases. The primary functions of tRNA methylations are linked to the different steps of protein synthesis, such as the stabilization of tRNA structure, reinforcement of the codon-anticodon interaction, regulation of wobble base pairing, and prevention of frameshift errors. However, beyond these basic functions, recent studies have demonstrated that tRNA methylations are also involved in the RNA quality control system and regulation of tRNA localization in the cell. In a thermophilic eubacterium, tRNA modifications and the modification enzymes form a network that responses to temperature changes. Furthermore, several modifications are involved in genetic diseases, infections, and the immune response. Moreover, structural, biochemical, and bioinformatics studies of tRNA methyltransferases have been clarifying the details of tRNA methyltransferases and have enabled these enzymes to be classified. In the final section, the evolution of modification enzymes is discussed.

## Introduction

The first tRNA sequence was determined in 1965 and numerous modifications were identified at various positions within the sequence (Holley et al., 1965). At almost the same time, several tRNA methyltransferase activities were detected in *Escherichia coli* cell extract (Hurwitz et al., 1964), which suggested that diverse enzymes are involved in tRNA modification. To date, more than 90 modified nucleosides have been identified in tRNA (Machnicka et al., 2013). Thus, the majority of modified nucleosides that have been discovered in different RNA species are found in tRNA. In the twenty-first century, the major modification pathways of tRNA have been elucidated on the basis of genome sequence data. These studies have demonstrated that the pathways of tRNA modification show diversity among living organisms. In this review, I focus on the methylated nucleosides in tRNA, together with tRNA methyltransferases, and introduce their basic roles as well as their more complex functions.

## The primary role of tRNA modifications is the regulation of protein synthesis

**Figure 1:**
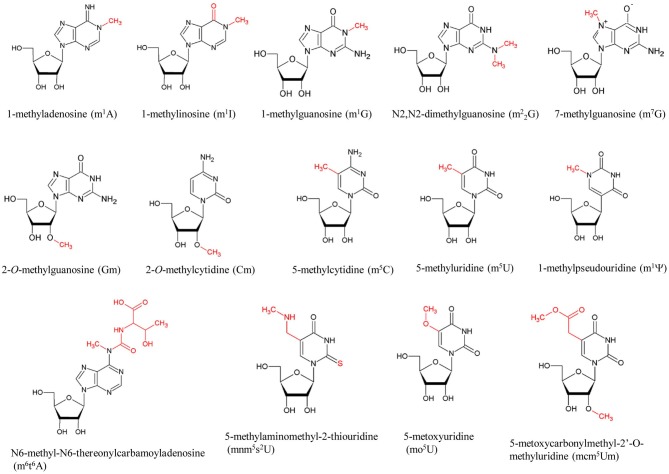
**Typical methylated nucleosides in tRNA**. The modification sites are colored in red. The abbreviations of the modified nucleosides are shown in parentheses.

**Figure 2:**
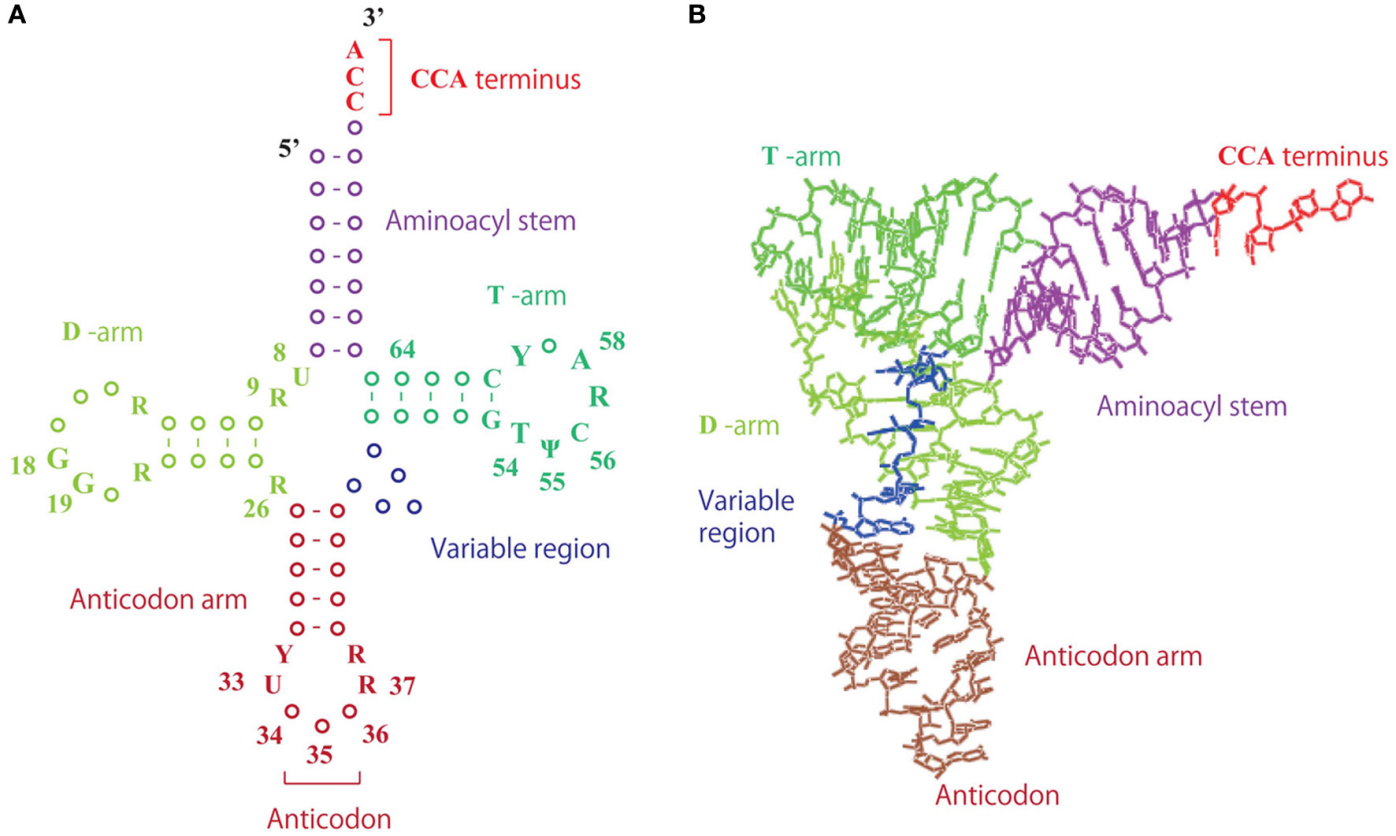
**Structure of tRNA. (A)** Clover-leaf structure of tRNA. The numbers show the positions of the nucleotides. **(B)** L-shaped tRNA structure. Transfer RNA forms an L-shaped structure, in which the D- and T-arms interact by tertiary base pairs.

Transfer RNA is an adaptor molecule that enables the genetic code of nucleic acids to be converted to amino acids in protein. Consequently, the primary functions of individual tRNA modifications are linked to the different steps of protein synthesis. In fact, if a tRNA remains unmodified, it becomes charged with a non-cognate amino acid, the corresponding codon in the mRNA is mistranslated, and a mutation is introduced. Table 1 summarizes the typical methylated nucleosides and their positions within the tRNA, their distributions in the three domains of life, the corresponding tRNA methyltransferases, their contributions to tRNA structure, their functions in addition to structural roles, and related publications. (Phillips and Kjellin-Straby, 1967; Taya and Nishimura, 1973; Yaniv and Folk, 1975; Delk et al., 1976; Watanabe et al., 1976, 2005, 2006; Pierre et al., 1978, 2003; Pope et al., 1978; Raba et al., 1979; Greenberg and Dudock, 1980; Ny and Bjork, 1980; Osorio-Almeida et al., 1980; Byström and Björk, 1982; Hopper et al., 1982; Walker, 1983; Gupta, 1984; Johnson et al., 1985; Ellis et al., 1986; Reinhart et al., 1986; van Tol et al., 1987; Ny et al., 1988; Björk et al., 1989, 2001; Jakab et al., 1990; Keith et al., 1990; Perret et al., 1990; Edmonds et al., 1991; Gu and Santi, 1991; Gustafsson and Björk, 1993; Hagervall et al., 1993; Edqvist et al., 1994; Kowalak et al., 1994; Martin and Hopper, 1994; Grosjean et al., 1995, 1996, 2008; Durand et al., 1997; Jiang et al., 1997; Li et al., 1997; Persson et al., 1997, 1998; Anderson et al., 1998, 2000; Constantinesco et al., 1998, 1999a,b; Helm et al., 1998; Hori et al., 1998, 2002, 2003; Matsuyama et al., 1998; Qian et al., 1998; Tomita et al., 1998; Cavaillé et al., 1999; Farabaugh and Björk, 1999; Liu et al., 1999, 2003, 2013; Motorin and Grosjean, 1999; Niederberger et al., 1999; Liu and Straby, 2000; Nordlund et al., 2000; Clouet-d'Orval et al., 2001, 2005; Dong et al., 2001; Urbonavicius et al., 2001, 2002, 2003, 2005; Yasukawa et al., 2001; Alexandrov et al., 2002, 2005, 2006; Johansson and Byström, 2002; King and Redman, 2002; Pintard et al., 2002; Suzuki et al., 2002, 2007, 2011a; Ahn et al., 2003; Bortolin et al., 2003; De Bie et al., 2003; Droogmans et al., 2003; Elkins et al., 2003; Jackman et al., 2003; Kalhor and Clarke, 2003; Kaneko et al., 2003; Kierzek and Kierzek, 2003; Takai and Yokoyama, 2003; Armengaud et al., 2004; Brulé et al., 2004; Bujnicki et al., 2004; Christian et al., 2004, 2013; Freude et al., 2004; Kadaba et al., 2004; Nasvall et al., 2004; Nureki et al., 2004; O'Dwyer et al., 2004; Okamoto et al., 2004; Roovers et al., 2004, 2008a,b, 2012; Singh et al., 2004; Cartlidge et al., 2005; Chen et al., 2005, 2011; Durant et al., 2005; Huang et al., 2005; Kalhor et al., 2005; Kirino et al., 2005; Leipuviene and Björk, 2005; Lu et al., 2005; Pleshe et al., 2005; Purushothaman et al., 2005; Renalier et al., 2005; Sakurai et al., 2005a,b; Umeda et al., 2005; Waas et al., 2005, 2007; Brzezicha et al., 2006; Goll et al., 2006; McCrate et al., 2006; Noma et al., 2006, 2010, 2011; Ote et al., 2006; Purta et al., 2006; Shigi et al., 2006; Takano et al., 2006; Takeda et al., 2006; Yim et al., 2006; Zegers et al., 2006; Auxilien et al., 2007, 2011, 2012; Begley et al., 2007; Christian and Hou, 2007; Choudhury et al., 2007a,b; Lee et al., 2007; Matsumoto et al., 2007; Ozanick et al., 2007; Walbott et al., 2007; Wilkinson et al., 2007; Alian et al., 2008; Barraud et al., 2008; Chernyakov et al., 2008; Goto-Ito et al., 2008, 2009; Ihsanawati et al., 2008; Klassen et al., 2008; Kurata et al., 2008; Kuratani et al., 2008, 2010; Leulliot et al., 2008; Meyer et al., 2008, 2009; Tomikawa et al., 2008, 2010, 2013; Toyooka et al., 2008; Awai et al., 2009, 2011; Lai et al., 2009; Moukadiri et al., 2009, 2014; Nishimasu et al., 2009; Osawa et al., 2009; Shi et al., 2009; Shimada et al., 2009; Umitsu et al., 2009; Ye et al., 2009; Arragain et al., 2010; Atta et al., 2010, 2012; Benítez-Páez et al., 2010; Böhme et al., 2010; Chen and Yuan, 2010; de Crécy-Lagard et al., 2010; Fu et al., 2010; Guelorget et al., 2010, 2011; Kempenaers et al., 2010; Mazauric et al., 2010; Ochi et al., 2010, 2013; Songe-Møller et al., 2010; Tkaczuk, 2010; D'Silva et al., 2011; Hamdane et al., 2011a,b, 2012, 2013; Joardar et al., 2011; Kitamura et al., 2011, 2012; Leihne et al., 2011; Liger et al., 2011; Lin et al., 2011; Menezes et al., 2011; Pearson and Carell, 2011; Qiu et al., 2011; van den Born et al., 2011; Wei et al., 2011; Armengod et al., 2012; Chan et al., 2012; Chatterjee et al., 2012; Chujo and Suzuki, 2012; Dewe et al., 2012; Fislage et al., 2012; Gehrig et al., 2012; Guy et al., 2012; Jöckel et al., 2012; Novoa et al., 2012; Pastore et al., 2012; Patil et al., 2012a,b; Perche-Letuvée et al., 2012; Sakaguchi et al., 2012; Towns and Begley, 2012; Vilardo et al., 2012; Wurm et al., 2012; Yamagami et al., 2012; Edelheit et al., 2013; Fujimori, 2013; Igoillo-Esteve et al., 2013; Kim and Almo, 2013; Ohira et al., 2013; Paris et al., 2013; Preston et al., 2013; Shao et al., 2013; Swinehart et al., 2013). In Table 1, several important tRNA modifications such as pseudouridine (ψ), lysidine, agmatidine, queosine (Q), and 2-thiouridine (s^2^U) are not listed because their biosynthetic pathways do not include any methylation steps. Nevertheless, Table 1 outlines the roles of key tRNA modifications, and demonstrates that methylated nucleosides and tRNA methyltransferases are very important for such functions. The structures of typical methylated nucleosides are shown in Figure 1. It is impossible to depict all methylated nucleosides in Figure 1 due to limitations of space. Please visit the database (http://modomics.genesilico.pl/modifications/) to obtain additional structural information (Machnicka et al., 2013). The structure of tRNA and positions of the methylated nucleotides are shown in Figure 2. As for tRNA stabilization by methylated nucleosides, see this review (Motorin and Helm, 2010). Even today, the contributions to tRNA structure and/or function in protein synthesis of many methylated nucleosides remain unknown (Table 1). However, various tRNA methyltransferases and their corresponding disruptant strains have been analyzed, and their functions are gradually being elucidated. Among the phenotypes of the gene disruptant strains, many phenomena have been reported that are difficult to understand directly in terms of enzyme function or effects on protein synthesis. For example, *E. coli* miaA mutant strains, which contain A37 instead of ms^2^i^6^A37 in the tRNA, show a moderate mutator phenotype that results in an increased rate of GC -> AT transversion (Zhao et al., 2001). Furthermore, inosine 34 modification in fission yeast is essential for cell cycle progression (Tsutsumi et al., 2007). These phenomena might be caused by changes in the amount of certain protein(s), such as transcription factors, in the disruptant strains. In fact, recently, it has been reported that Trm9-specific tRNA modifications enhance codon-specific elongation of translation and promote increased levels of DNA damage response proteins (Begley et al., 2007). Furthermore, several eukaryotic tRNA methyltransferases (for example, human ALKBH8 Shimada et al., 2009; Fu et al., 2010 and yeast Trm2 Choudhury et al., 2007a,b) are involved directly in DNA repair and carcinogenesis because they exist as fusion proteins with other enzyme(s). However, it remains possible that some of the phenotypes observed in the disruptant strains are linked to unknown biological phenomena.

**Table 1:** **Typical methylated nucleosides in tRNA and corresponding tRNA methyltransferases**.

**Name and position**	**Distribution**	**Methyltransferase(s)**	**Contribution to tRNA structure**	**Function(s) in addition to structural role**	**References**
Am4 and Cm4	E	Trm13	Stabilization of aminoacyl stem?		Wilkinson et al., 2007; Tkaczuk, 2010
Am6	A (*Pyrococcus furiosus*)	?	Stabilization of aminoacyl stem?		Constantinesco et al., 1999a
m^2^G6	E/B/A	?/TrmN/Trm14	Stabilization of aminoacyl stem?		Menezes et al., 2011; Fislage et al., 2012; Roovers et al., 2012
m^2^G7	E	?	Stabilization of aminoacyl stem?	Related to Sarcoma-virus infection	Pierre et al., 1978
m^1^G9 and m^1^A9	E (mitochondria)	MRPP1	Correct folding of mitochondrial tRNA	Complex formation with mitochondrial RNase P.	Helm et al., 1998; Sakurai et al., 2005a; Vilardo et al., 2012
				Marker of processing of 5′-leader sequence?	
				Recognition site for Nematoda mitochondrial EF-Tu2	
	A	Archaeal Trm10 homolog			Kempenaers et al., 2010
m^1^G9	E	Trm10	Defect causes young onset diabetes in humans.		Jackman et al., 2003; Igoillo-Esteve et al., 2013; Shao et al., 2013; Swinehart et al., 2013
m^2^G10	E	Trm11 and Trm112 complex.			Purushothaman et al., 2005
m^2^_2_G10	A	Archaeal Trm-m^2^_2_G10 enzyme	Prevention of alternative tRNA structure		Armengaud et al., 2004; Urbonavicius et al., 2006
Gm18	E/B	Trm3/TrmH	Stabilization of D-arm and T-arm interaction		
			Stabilization of L-shaped tRNA	Please see main text	Persson et al., 1997; Hori et al., 1998, 2002, 2003; Cavaillé et al., 1999; Urbonavicius et al., 2002; Nureki et al., 2004; Pleshe et al., 2005; Watanabe et al., 2005, 2006; Ochi et al., 2010, 2013; Gehrig et al., 2012; Jöckel et al., 2012
m^1^A22	B	TrmK	Prevention of Watson-Crick base pair formation?		Roovers et al., 2008a
m^2^_2_G26 (m^2^G26)	E/A	Trm1			
	Trm1 transfers two methyl groups to G26, so m^2^G26 is produced as an intermediate				
	Eukaryotic Trm1 localizes to both the nuclear membrane and mitochondria				
			Prevention of Watson-Crick base pair formation?		
			Stabilization of the three-dimensional core?		Phillips and Kjellin-Straby, 1967; Hopper et al., 1982; Ellis et al., 1986; Reinhart et al., 1986; Edqvist et al., 1994; Martin and Hopper, 1994; Constantinesco et al., 1998, 1999b; Liu et al., 1999; Niederberger et al., 1999; Liu and Straby, 2000; Grosjean et al., 2008; Ihsanawati et al., 2008; Lai et al., 2009; D'Silva et al., 2011; Dewe et al., 2012
m^2^_2_G26 (m^2^G26) and m^2^_2_G27 (m^2^G27)	E?/B	Trm1			
	*Aquifex aeolicus* Trm1 modifies G27 as well as G26				
	The modification pattern of tRNAs suggests that some mammalian Trm1 enzymes might act on both G26 and G27 similar to *A. aeolicus* Trm1				
			Prevention of Watson-Crick base pair formation?		
			Stabilization of the three-dimensional core?		
			In the case of G27 modification, stabilization of the anticodon arm?		Johnson et al., 1985; van Tol et al., 1987; Awai et al., 2009, 2011
m^5^C27	E	?			
	This modification is identified by the bisulfite method: the modification might be an m^5^C derivative				Edelheit et al., 2013
Cm32 and Um32	B	TrmJ	Stabilization of the anticodon loop		Purta et al., 2006
Cm32	A	?			
	Cm32 modification is observed in several tRNA species from *H. volcanii* and *T. acidophilum*				
			Stabilization of the anticodon loop		Walker, 1983; Gupta, 1984
Cm32 and Nm34	E	Trm7 and Trm732 complex synthesizes Cm32			
(Cm34, Gm34, and ncm^5^Um34)	Trm7 and Trm734 complex synthesizes Nm34				
			Stabilization of the anticodon-loop	In the case of Nm34, reinforcement of the codon-anticodon iteraction.	
				Cm32 and Gm34 in tRNA^Phe^ are required for efficient yW37 formation.	
				FTSJ1 (human TRM7) is implicated in nonsyndromic X-linked mental retardation	Pintard et al., 2002; Freude et al., 2004; Guy et al., 2012
m^3^C32	E/A	Trm140/?	Stabilization of the anticodon-loop?	The yeast *trm140* -*trm1* double knockout strain is sensitive to low concentrations of cycloheximide	D'Silva et al., 2011; Noma et al., 2011
Cm34 and cmnm^5^Um34	B	TrmL	Stabilization of the anticodon-loop.	Reinforcement of the codon-anticodon interaction	Benítez-Páez et al., 2010; Liu et al., 2013
Cm34 and Um39	A	Complex of aFib, Nop5p and L7Ae with box C/D guide RNA (intron)			
	Cm34 and Um39 in tRNA^Trp^ from *P. abyssi* and *H. volcanii* are introduced by the box C/D ribonucleoprotein and guide RNA system				
	The guide RNA is an intron in precursor-tRNA^Trp^				
	Several 2′-O-methylations in archaeal tRNAs are predicted to be formed by the box C/D ribonucleoprotein and guide RNA system				
			Stabilization of the anticodon-arm?	Reinforcement of the codon-anticodon interaction (Cm34)	Clouet-d'Orval et al., 2001; Bortolin et al., 2003; Singh et al., 2004; Clouet-d'Orval et al., 2005; Ye et al., 2009; Lin et al., 2011
Cm34	A (Haloarchaea)	Complex of aFib, Nop5p and L7Ae with box C/D guide RNA (sR-tMet)			
				Reinforcement of the codon-anticodon interaction	Joardar et al., 2011
Xm^5^U34 derivatives	E/B/A				
	Biosynthetic pathways of Xm^5^U34 derivatives are not completely clarified				
	For information on the outlines of Xm^5^U34 biosynthesis pathways, please see these references,				
	Umeda et al., 2005; Chen et al., 2011; van den Born et al., 2011; Moukadiri et al., 2014				
	In some cases, methylation by tRNA methyltransferases is part of the multistep reactions				
	MnmE and MnmG complex and MnmC generates mnm^5^U34				
	*Aquifex aeolicus* DUF752 protein is a tRNA methyltransferase that functions without the usually fused oxidase domain				
	The Trm9-Trm112 complex forms mcm^5^U34 from cm^5^U34				
	The Trm9 homolog in mammalians, *C. elegans* and plants is a methyltransferase domain of ALKBH8				
	The Alk domain in ALKBH8 stereoselectively generates S-mchm^5^U34 from mcm^5^U34				
	Human MTO1, MSS1 and MTU1 are involved in τm^5^s^2^U34 formation in mitochondrial tRNA				
			Stabilization of the anticodon loop	Reinforcement of the codon-anticodon interaction, restriction of wobble base pairing, and prevention of frameshift error	
				Transfer RNAs with the mcm^5^U modification are the target of *Kluyveromyces lactis* gamma-toxin and *Pichia acaciae* killer toxin	
				Trm9-specific tRNA modifications enhance codon-specific translational elongation and promote increased levels of DNA damage response proteins. The synthesized DNA damage response proteins affect with cell cycle regulation	
				ALKBH8 is involved in DNA repair and carcinogenesis	
				Lack of τm^5^s^2^U34 in human mitochondrial tRNA^Lys^ causes myoclonus epilepsy associated with ragged-red fibers	Taya and Nishimura, 1973; Keith et al., 1990; Urbonavicius et al., 2001, 2003; Yasukawa et al., 2001; Suzuki et al., 2002, 2011a; Kalhor and Clarke, 2003; Kaneko et al., 2003; Takai and Yokoyama, 2003; Bujnicki et al., 2004; Chen et al., 2005, 2011; Huang et al., 2005; Kirino et al., 2005; Leipuviene and Björk, 2005; Lu et al., 2005; Sakurai et al., 2005b; Umeda et al., 2005; Yim et al., 2006; Begley et al., 2007; Klassen et al., 2008; Kurata et al., 2008; Meyer et al., 2008, 2009; Roovers et al., 2008b; Moukadiri et al., 2009, 2014; Osawa et al., 2009; Shi et al., 2009; Shimada et al., 2009; Böhme et al., 2010; Fu et al., 2010; Mazauric et al., 2010; Songe-Møller et al., 2010; Kitamura et al., 2011, 2012; Leihne et al., 2011
					Liger et al., 2011; Pearson and Carell, 2011; van den Born et al., 2011; Armengod et al., 2012; Pastore et al., 2012; Patil et al., 2012a,b; Kim and Almo, 2013; Ohira et al., 2013; Tomikawa et al., 2013
Xmo^5^U34 derivatives	B	?		Expansion of wobble base pairing	Pope et al., 1978; Nasvall et al., 2004; Novoa et al., 2012
m^7^G34	E (Mitochondria)	?		Expansion of wobble base pairing?	Matsuyama et al., 1998; Tomita et al., 1998
m^7^G36	E (Chloroplast)	?			Osorio-Almeida et al., 1980; Jakab et al., 1990
m^1^G37	E/B/A	Trm5/TrmD/Trm5		Prevention of frameshift errors.	
				Prevention of misacylation of tRNA^Asp^ by Arg-RS	Osorio-Almeida et al., 1980; Byström and Björk, 1982; Björk et al., 1989, 2001; Perret et al., 1990; Hagervall et al., 1993; Li et al., 1997; Farabaugh and Björk, 1999; Ahn et al., 2003; Elkins et al., 2003; Liu et al., 2003; Brulé et al., 2004; Christian et al., 2004, 2013; O'Dwyer et al., 2004; Takeda et al., 2006; Christian and Hou, 2007; Lee et al., 2007; Goto-Ito et al., 2008, 2009; Toyooka et al., 2008; Sakaguchi et al., 2012; Paris et al., 2013
m^1^I37	E	Trm5			
	The m^1^I37 modification is often observed in eukaryotic tRNA^Ala^ (for example, yeast tRNA^Ala^)				
	The m^1^I37 is synthesized from A37 *via* I37.				Holley et al., 1965; Grosjean et al., 1996; Brulé et al., 2004
yW37 derivatives	E/A	Trm5 + Tyw3/Trm5 (homologs) + Taw3			
	For information on the biosynthetic pathways of yW37 derivatives, please see these references, Noma et al., 2010; de Crécy-Lagard et al., 2010				
	Biosynthesis of yW37 derivatives starts with the m^1^G37 modification by Trm5 in both eukaryotes and archaea				
	Some archaeal biosynthetic pathways are predicted from genomic information				
	In some intermediate steps, methylation(s) by Tyw3 (yeast) or Taw3 and Trm5 homologs (archaea) and a radical SAM reaction are involved				
	The names, Trm12 and TrmM, were previously allocated to eukaryotic and archaeal methyltransferases, respectively in yW37 biosynthetic pathways				
	Therefore, some genome sequence projects used these names				
			Stabilization of the anticodon loop		
				Prevention of frameshift errors	Jiang et al., 1997; Kalhor et al., 2005; Waas et al., 2005, 2007; Noma et al., 2006, 2010; Suzuki et al., 2007; Umitsu et al., 2009; de Crécy-Lagard et al., 2010; Perche-Letuvée et al., 2012
t^6^A37 derivatives	E/B/A				
	The biosynthetic pathway of m^6^t^6^A contains a methylation step				
	TsaA is involved in the methylation in the m^6^t^6^A modification				
	MtaB is a methylthiotransferase for ms^2^t^6^A formation (a radical SAM enzyme)				
	Mammalian Cdkal1 is a radical SAM-enzyme that forms ms^2^t^6^A in tRNA^Lys^				
			Stabilization of the anticodon loop.	Prevention of frameshift errors	
				A defect of ms^2^t^6^A in tRNA^Lys^ causes type 2 diabetes in mice	Gupta, 1984; Qian et al., 1998; Durant et al., 2005; McCrate et al., 2006; Arragain et al., 2010; Atta et al., 2010, 2012; Wei et al., 2011; Fujimori, 2013
i^6^A37 derivatives	B				
	The 2-methyltio group of ms^2^i^6^A derivatives is formed by MiaB (a radical SAM enzyme)				
			Stabilization of the anticodon loop.	Prevention of frameshift errors	
				Hydroxylation of ms^2^io^6^A37 is related to utilization of TCA cycle products	
				*Shigella flexneri* MiaA is required for expression of virulence genes	Durand et al., 1997; Li et al., 1997; Persson et al., 1998; Farabaugh and Björk, 1999; Urbonavicius et al., 2001, 2003; Kierzek and Kierzek, 2003; Pierre et al., 2003; Ote et al., 2006; Atta et al., 2010, 2012; Fujimori, 2013
m^2^A37	B	?			Yaniv and Folk, 1975
m^6^A37	B	TrmG?			Qian et al., 1998
m^5^C38	E/B?	Dnmt2			
	Dnmt2 is a methyltransferase with high sequence similarity to DNA methyltransferases				Dong et al., 2001; Goll et al., 2006
m^5^C34, m^5^C40, m^5^C48 and m^5^C49	E/A	Trm4 (human Misu)/Trm4			
	Site specificities of tRNA m^5^C methyltransferases are not completely clarified				
	Trm4 homologs might be involved in the methylation(s) of other position(s)				
	m^5^C34 and m^5^C40 in yeast tRNAs are introduced in an intron-dependent manner				
	Archease binds to archaeal Tm4 and regulates the specificity of methylation site				
	Human Trm4 (Misu) catalyzes the m^5^C34 formation in tRNA^Leu^ in an intron-dependent manner				
	Recently, it has been reported that human Trm4 is multi-site specific				
			Stabilization of the three-dimensional core?		
				Under oxidative stress, yeast tRNA^Leu^ changes the level of m^5^C modifications which lead to selective translation of mRNA	
				The half-life of tRNA^Val^ is shortened in the yeast *trm8* and *trm4* double knockout strain	
				The level of m^5^C modification in tRNA^His^ increases in response to growth arrest in *S. cerevisiae*	Gupta, 1984; Motorin and Grosjean, 1999; King and Redman, 2002; Alexandrov et al., 2006; Brzezicha et al., 2006; Auxilien et al., 2007, 2012; Walbott et al., 2007; Chernyakov et al., 2008; Kuratani et al., 2010; Chan et al., 2012; Dewe et al., 2012; Edelheit et al., 2013; Preston et al., 2013
m^7^G46	E/B	Trm8 (human METTL1)-Trm82 complex/TrmB.	Stabilization of the three-dimensional core?		
				Half-life of tRNA^Val^ is shortened in the yeast *trm8* and *trm4* double knockout strain	
				Gene disruption of Trm8 homolog in *Colletotrichum lagenarium* causes the loss of infectious ability	
				In the case of *T. thermophilus*, m^7^G46 modification is one of the key factors in network of modified nucleotides and tRNA modification enzymes and is essential for growth at high temperatures. Please see main text	Alexandrov et al., 2002, 2005, 2006; De Bie et al., 2003; Okamoto et al., 2004; Cartlidge et al., 2005; Takano et al., 2006; Zegers et al., 2006; Matsumoto et al., 2007; Chernyakov et al., 2008; Leulliot et al., 2008; Tomikawa et al., 2008, 2010; Dewe et al., 2012
m^7^G49	A	?			
	*T. acidophilum* tRNA^Leu^ exceptionally has the m^7^G49 modification				Edmonds et al., 1991; Tomikawa et al., 2013
m^5^C51	A	?			Auxilien et al., 2007
m^5^C52	A	?			Auxilien et al., 2007
m^5^U54 derivatives	E/B/A (*Pyrococcus furiosus* and *Pyrococcus abyssi*)	Trm2 + a/TrmA or TrmFO/RlmD-like protein (PA0719)			
	The m^5^U54 modification in some gram-negative bacteria including *E. coli* is synthesized by TrmA				
	The m^5^U54 modification in gram-positive and some gram-negative bacteria is synthesized by TrmFO				
	*Pyrococcus abyssi* rRNA methyltransferase (RlmD)-like protein synthesizes m^5^U54				
	In thermophilic eubacteria and archaea, m^5^U54 is further modified to m^5^s^2^U54				
	In mammalian tRNA^Lys^, U54 is probably modified to m^5^Um54 *via* m^5^U54: the second methyltransferase has not been identified				
			Formation of the reverse Hoogsteen base pair with A58		
			Stabilization of the T-loop structure		
			Stabilization of the T-arm and D-arm interaction		
				*E. coli* TrmA binds to rRNA and this binding is essential for cell viability	
				Eukaryotic Trm2 has a 5′ -> 3′ endonuclease activity and is involved in DNA repair	Delk et al., 1976; Watanabe et al., 1976; Raba et al., 1979; Greenberg and Dudock, 1980; Ny and Bjork, 1980; Ny et al., 1988; Edmonds et al., 1991; Gu and Santi, 1991; Gustafsson and Björk, 1993; Kowalak et al., 1994; Constantinesco et al., 1999a; Nordlund et al., 2000; Johansson and Byström, 2002; Urbonavicius et al., 2002, 2005; Shigi et al., 2006; Choudhury et al., 2007a,b; Matsumoto et al., 2007; Alian et al., 2008; Leulliot et al., 2008; Tomikawa et al., 2008, 2010; Awai et al., 2009; Nishimasu et al., 2009; Auxilien et al., 2011; Hamdane et al., 2011a,b, 2012, 2013; Yamagami et al., 2012
m^1^ψ54 derivatives	A	TrmY [Mja 1640 (*Methanocaldococcus jannaschii*), and Hvo 1989 (*Haloferax volcanii*)]			
			Formation of the reverse Hoogsteen base pair with A58?		
			Stabilization of the T-loop structure?		
			Stabilization of the T-arm and D-arm interaction?		Gupta, 1984; Chen and Yuan, 2010; Chatterjee et al., 2012; Wurm et al., 2012
Cm56	A	aTrm56 or the complex of aFib, Nop5p and L7Ae with BoxC/D guide RNA (*Pyrobaculum aerophylum*)			
			Stabilization of the T-loop structure?		
			Stabilization of the T-arm and D-arm interaction?		Walker, 1983; Gupta, 1984; Constantinesco et al., 1999a; Renalier et al., 2005; Kuratani et al., 2008; Tomikawa et al., 2013
m^5^C56	E	?			
		This modification is identified by the bisulfite method: the modification might be a m^5^C derivative			Edelheit et al., 2013
m^1^A57 and m^1^A58	A	TrmI (aTrmI)	Formation of the reverse Hoogsteen base pair between m^5^U54 and m^1^A58		
			Stabilization of the T-loop structure		Walker, 1983; Gupta, 1984; Constantinesco et al., 1999a; Roovers et al., 2004; Guelorget et al., 2010, 2011; Tomikawa et al., 2013
m^1^I57	A	TrmI (aTrmI)			
		m^1^I57 is formed from m^1^A57 by deamination			Walker, 1983; Gupta, 1984; Grosjean et al., 1995
m^2^G57	A	?			Walker, 1983
m^1^A58	E/B	Trm6-Trm61 complex, and Trmt61B (Mitochondria)/TrmI			
			Formation of the reverse Hoogsteen base pair between m^5^U54 and m^1^A58		
			Stabilization of the T-loop structure		
				The m^1^A58 modification in *S. cerevisiae* initiator tRNA^Met^ functions in the RNA quality control system	
				The m^1^A58 modification in *T. thermophilus* tRNA is required for cell growth at high temperatures. Please see main text	Anderson et al., 1998, 2000; Droogmans et al., 2003; Kadaba et al., 2004; Ozanick et al., 2007; Barraud et al., 2008; Guelorget et al., 2010; Qiu et al., 2011; Chujo and Suzuki, 2012

This table focuses on the methylated nucleosides in tRNA and tRNA methyltransferases. Consequently, the detailed biosynthetic pathways of complicated modified nucleosides that do not involve methylation are not explained. They are summarized as “derivatives.” Although many methylated nucleosides and their methyltransferases have been studied for more than 40 years, recent publications are mainly cited in the references due to limitation of space. Abbreviations are as follows: Eukaryote, E; Eubacteria, B; Archaea, A. The eukaryotic enzyme names are based on the yeast enzyme names. For human enzymes, please see this review Towns and Begley (2012).

## Multiple regulation of tRNA modification pathways and importance of the availability of methyl donors

In living cells, more than 50% of the high energy compounds such as ATP, that are produced by respiration are consumed by protein synthesis. Furthermore, the most important metabolic pathway of amino acids is protein synthesis. The metabolic pathways of energy and amino acids are closely linked. Studies on the pathways of tRNA modification have revealed that the RNA modification systems are located downstream of the pathways of energy and amino acid metabolism and that they are regulated at multiple steps (Herbig et al., 2002; Iwata-Reuyl, 2003; Ikeuchi et al., 2008, 2010; Shigi et al., 2008; Suzuki and Miyauchi, 2010; Phillips et al., 2012; Laxman et al., 2013; Miyauchi et al., 2013; Perrochia et al., 2013 Figure 3 and Table 1). Thus, depletion of a certain compound (for example, one of the amino acids) or disruption of a metabolic pathway can result in incomplete modification of tRNA and thus an increased frequency of translational errors.

**Figure 3:**
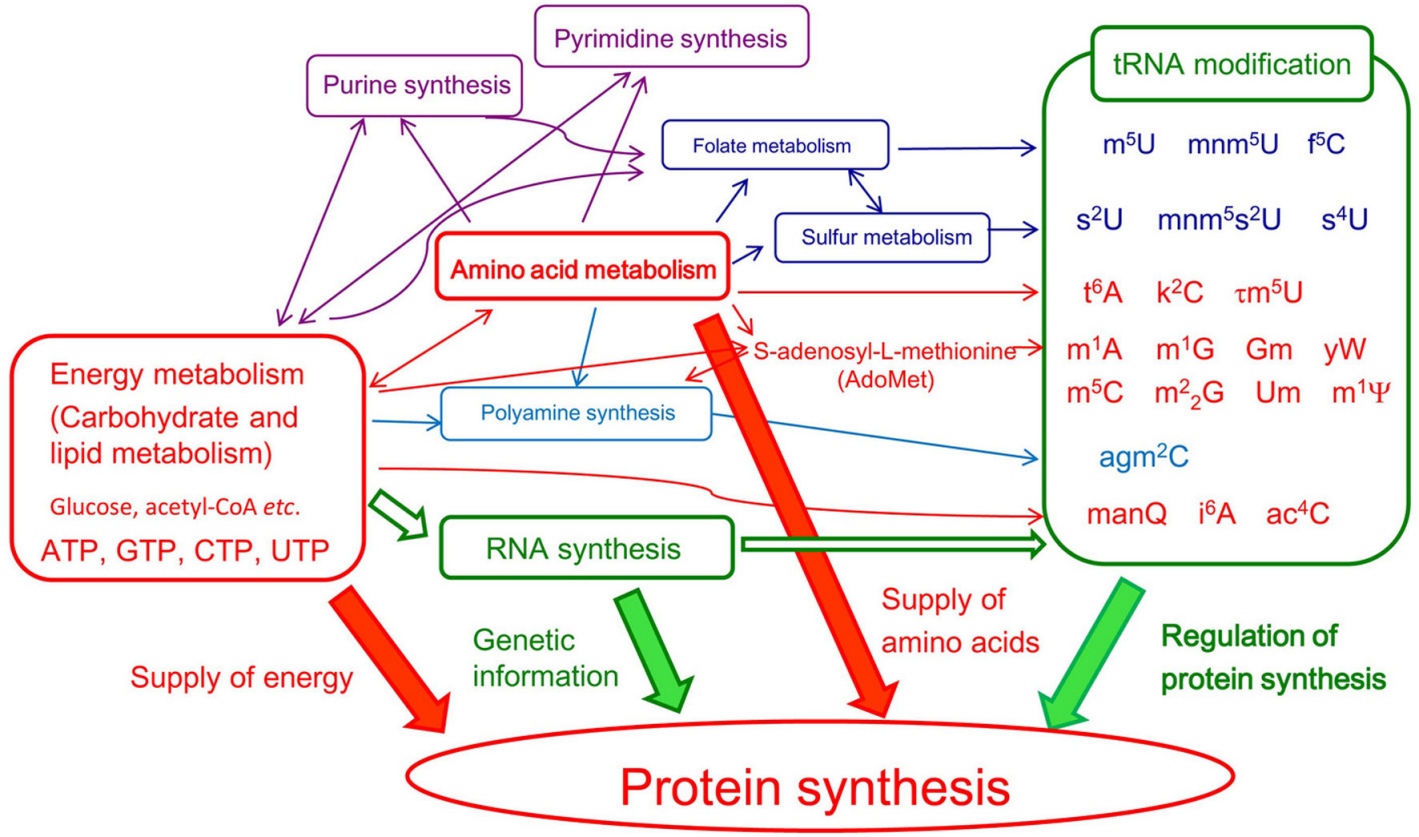
**Transfer RNA modifications are regulated by various metabolic pathways**. In this figure, conversions of chemical compounds and conceptual effects are shown by thin and thick arrows, respectively. To emphasis the relationships among energy metabolism, amino acid metabolism, tRNA modification, and protein synthesis, DNA replication and so on are omitted.

The structures of identified modified nucleosides suggest that the majority of tRNA modifications require a methylation step(s) (Table 1 and Figure 1). The methyl-transfer reaction by majority of tRNA methyltransferases consumes S-adenosyl-L-methionine (AdoMet) as the methyl-group donor. Thus, the depletion of AdoMet leads to multiple incomplete modifications in tRNA. The precursors of AdoMet are ATP and methionine. These facts seem to provide an answer for the question, “Why do living organisms use the methionine codon as the initiation codon for protein synthesis?” Under conditions where methionine is limited and the tRNA contains multiple incomplete modifications, to avoid increase of frequency of translational error, the methionine codon is selected the initiation codon of protein synthesis. Analogously, the fact that eubacterial methionyl-initiator tRNA^Met^ is formylated and formylation is the transfer of one carbon atom suggests that the supply of sources of single carbon atoms is very important for efficient and accurate protein synthesis in bacteria.

## Structures of tRNA methyltransferases

Transfer RNA methyltransferases can be divided into two types on the basis of their methyl donor: one class uses AdoMet whereas the other utilizes 5, 10-methylenetetrahydrofolate (Table 2). As mentioned above, the majority of tRNA methyltransferases are AdoMet-dependent. For information on the catalytic mechanisms of tRNA methyltransferases, (see Watanabe et al., 2005; Kuratani et al., 2008; Meyer et al., 2008; Osawa et al., 2009; Hou and Perona, 2010; Hamdane et al., 2012). Recently, a radical SAM enzyme was identified as a ribosomal RNA methyltransferase (Atta et al., 2010); radical SAM enzymes utilize a 4Fe-4S cluster to generate a reactive radical from AdoMet. No radical SAM enzymes that act as tRNA methyltransferases have been identified as yet. However, three types of radical SAM enzymes are involved in tRNA modifications (2-methylthiotransferases that generate ms^2^t^6^A derivatives, 2-methylthiotransferases that generate ms^2^i^6^A derivatives, and enzymes involved in the biosynthesis of yW37 derivatives) (Suzuki et al., 2007; Atta et al., 2010, 2012; de Crécy-Lagard et al., 2010; Fujimori, 2013 and Table 1). Radical SAM tRNA methyltransferase(s) might be identified in the near future, because there are many methylated nucleosides, for which the responsible enzyme(s) have not yet been identified (Table 1).

**Table 2:** **Classification of tRNA methyltransferases by crystal structures**.

**Name**	**References**
**S-ADENOSYL-L-METHIONINE-DEPENDENT ENZYMES**	
**Class I**	
TrmA	Alian et al., 2008
TrmB	Zegers et al., 2006
MnmC	Barraud et al., 2008; Kitamura et al., 2011
TrmI and aTrmI	Roovers et al., 2004; Guelorget et al., 2010, 2011
TrmN	Fislage et al., 2012
Trm1	Ihsanawati et al., 2008; Awai et al., 2011
Trm4	Kuratani et al., 2010
Trm5	Goto-Ito et al., 2008, 2009
Trm8–Trm82	Leulliot et al., 2008
Trm14	Fislage et al., 2012
AlkB homolog 8 (domains)	Pastore et al., 2012
Fibrillalin, Nop5 and L7Ae complex	Ye et al., 2009; Lin et al., 2011
Dnmt2	Dong et al., 2001
**Class IV**	
TrmD	Ahn et al., 2003; Elkins et al., 2003; Liu et al., 2003
TrmH	Nureki et al., 2004; Pleshe et al., 2005
TrmL (YibK)	Lim et al., 2003; Liu et al., 2013
TrmY	Chen and Yuan, 2010; Chatterjee et al., 2012; Wurm et al., 2012
Trm10	Shao et al., 2013
aTrm56	Kuratani et al., 2008
**Radical SAM-tRNA methyltransferase**	
Unknown	
**5, 10-METHYLENETETRAHYDROFOLATE-DEPENDENT ENZYMES**	
MnmG	Meyer et al., 2008; Osawa et al., 2009; Shi et al., 2009
TrmFO	Nishimasu et al., 2009

The enzymes, of which structures have been determined by X-ray crystal structure studies, are listed. There are various enzymes, of which structures have been predicted by their amino acid sequences, conserved motifs and bioinformatics studies (Gustafsson et al., 1996; Anantharaman et al., 2002; Purta et al., 2006; Roovers et al., 2008a; Phizicky and Hopper, 2010; Tkaczuk, 2010). Detailed insight into catalytic mechanisms of tRNA methyltransferases is only available in a few cases: see these references (Watanabe et al., 2005; Kuratani et al., 2008; Meyer et al., 2008; Osawa et al., 2009; Hou and Perona, 2010; Hamdane et al., 2012).

**Figure 4:**
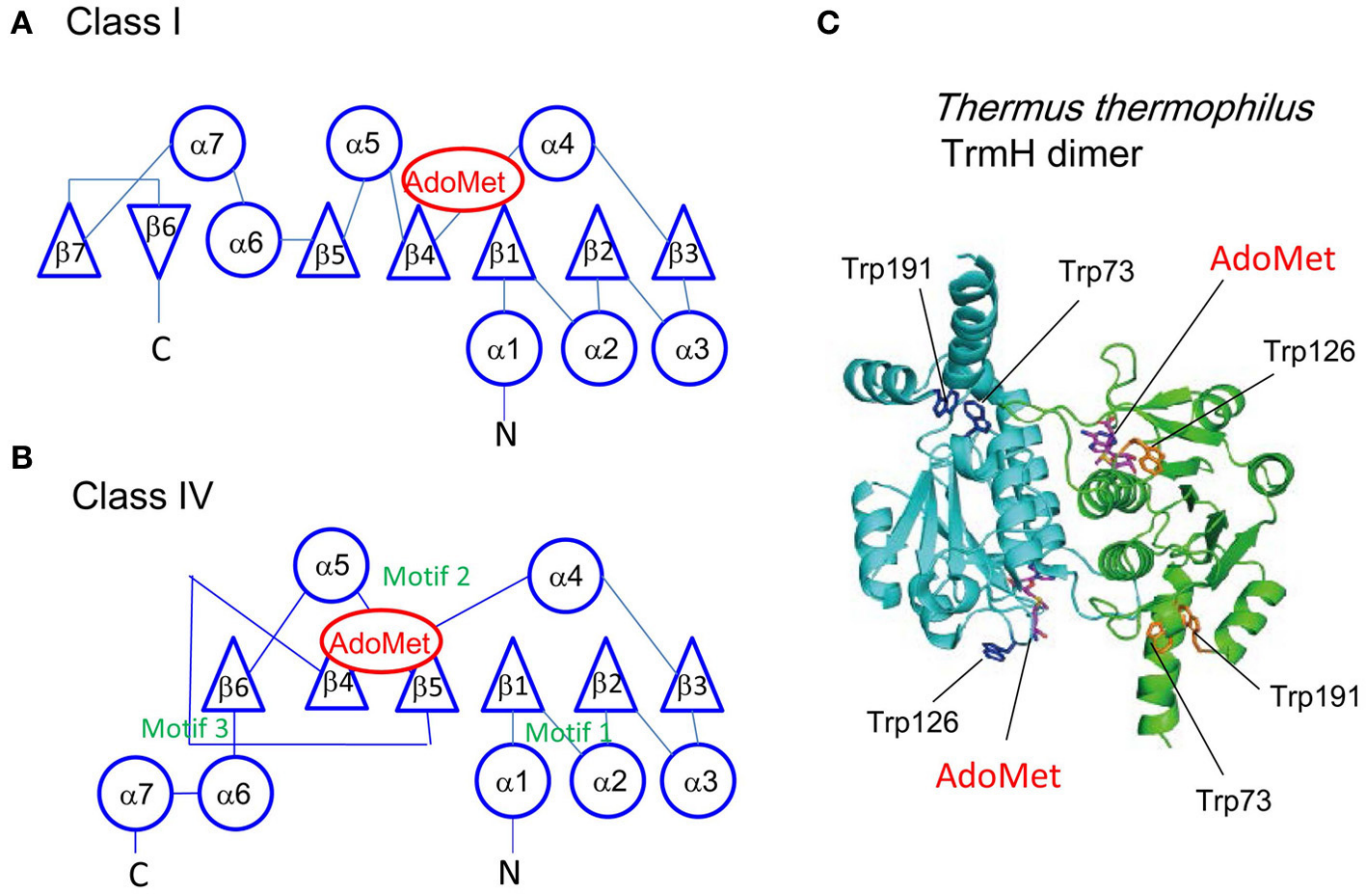
**Structures of Rossman fold (Class I) and topological knot fold (Class IV)**. The topologies of class I **(A)** and IV **(B)** folds are compared. Circles and triangles show α-helices and β-strands, respectively. The AdoMet binding sites and three conserved motifs in the class IV are shown in red and green, respectively. The known class IV enzymes work as a dimer. **(C)** The dimer structure of *T. thermophilus* TrmH. *T. thermophilus* TrmH is a typical class IV enzyme. Fluorescence derived from three tryptophan residues (Trp73, Trp126, and Trp191) was monitored in the stopped-flow pre-steady state kinetic analysis as described in the main text. This figure is based on these publications Clouet-d'Orval et al. (2005), Ochi et al. (2013) with slight modifications.

AdoMet-dependent methyltransferases are classified by their catalytic domain (Schubert et al., 2003). Two different classes (classes I and IV) have been identified among the tRNA methyltransferases (Table 2). Class I enzymes contain the Rossmann fold in the catalytic domain (Figure 4A), whereas class IV enzymes have the topological-knot structure (Figure 4B). Class IV enzymes were predicted initially by bioinformatics studies to be members of the SpoU-TrmD (SPOUT) superfamily (Anantharaman et al., 2002). Subsequently, crystallographic studies (Table 2) revealed that these enzymes have a topological knot structure. YibK was predicted initially to be an RNA methyltransferase of unknown function (Gustafsson et al., 1996). Determination of the crystal structure revealed the presence of the topological-knot structure in the catalytic domain of YibK (Lim et al., 2003). Later, YibK was shown to function as tRNA (Cm34/cmnm^5^Um34) methyltransferase and was renamed TrmL (Benítez-Páez et al., 2010; Liu et al., 2013). At almost the same time, three groups independently reported the crystal structures of TrmD proteins and revealed that TrmD proteins also contain the topological-knot structure (Ahn et al., 2003; Elkins et al., 2003; Liu et al., 2003). In 1997, SpoU was found to have tRNA (Gm18) 2′-*O*-methyltransferase activity and was renamed as TrmH (Persson et al., 1997). We solved the crystal structure of TrmH in 2004 and confirmed that it is a class IV enzyme with the topological-knot structure (Nureki et al., 2004 and Figure 4C). These studies established the structural foundation of SPOUT enzymes (Anantharaman et al., 2002; Tkaczuk et al., 2007), which can be identified on the basis of the topological-knot structure. To date, several tRNA methyltransferases have been identified as members of the SPOUT superfamily on the basis of crystal structures (Kuratani et al., 2008; Chen and Yuan, 2010; Chatterjee et al., 2012; Wurm et al., 2012; Shao et al., 2013) or structures predicted from amino acid sequences and conserved motifs (Renalier et al., 2005; Purta et al., 2006; Tkaczuk et al., 2007; Kempenaers et al., 2010, and Figure 4B). Furthermore, the SPOUT superfamily is expanding beyond the SpoU and TrmD families: novel enzymes such as an archaeal Trm10 homolog (Kempenaers et al., 2010) and TrmY (Chen and Yuan, 2010; Chatterjee et al., 2012; Wurm et al., 2012) have been identified. These enzymes cannot be simply classified into the SpoU or TrmD families. Therefore, it might be necessary to reclassify the enzymes of the SPOUT superfamily on the basis of their structure, the methylated nucleosides produced, and their reaction mechanisms.

The number of identified class I methyltransferases has also increased. Crystal structures of class I enzymes have been reported, as shown in Table 2; however, for many of the enzymes, structures have been predicted from their amino acid sequences and conserved motifs. The difficulty with crystallographic studies is that the eukaryotic and archaeal enzymes often require other subunit(s) to regulate (or stabilize) their activities (Anderson et al., 1998; Alexandrov et al., 2002, 2005; Purushothaman et al., 2005; Mazauric et al., 2010; Liger et al., 2011; Noma et al., 2011, and Table 1). Only a few structural studies of the multisubunit complexes have been performed, namely Trm8–Trm82 (Leulliot et al., 2008), and the Fibrillalin, Nop5 and L7Ae complex (Ye et al., 2009; Lin et al., 2011). In addition, structures for the tRNA bound-form of Trm5 (Goto-Ito et al., 2009) and T-arm-like RNA bound-form of TrmA (Alian et al., 2008) have been reported. Furthermore, several eukaryotic tRNA methyltransferases are fused with other functional domains and are involved in other processes such as DNA repair (Choudhury et al., 2007a,b; Shimada et al., 2009; Fu et al., 2010; Songe-Møller et al., 2010; D'Silva et al., 2011; Leihne et al., 2011; Noma et al., 2011; van den Born et al., 2011; Pastore et al., 2012). Although the crystal structures of the RNA recognition motif and AlkB domains of ALKB8H, which also contains a methyltransferase domain, have been reported (Pastore et al., 2012), there is no entire crystal structure of a eukaryotic multidomain tRNA methyltransferase. To understand the reaction mechanisms, substrate specificity, subunit (domain) interactions, and regulation of activity of these enzymes, structural studies are necessary.

Among the enzyme complexes that are involved in tRNA methylation, the mnmEG and mnmC complexes, which are required for the mnm^5^U34 modification (Taya and Nishimura, 1973; Bujnicki et al., 2004; Yim et al., 2006; Meyer et al., 2008, 2009; Roovers et al., 2008b; Moukadiri et al., 2009, 2014; Osawa et al., 2009; Shi et al., 2009; Böhme et al., 2010; Kitamura et al., 2011, 2012; Pearson and Carell, 2011; Armengod et al., 2012; Kim and Almo, 2013), are only found in eubacteria, which shows the complexity of the Xm^5^U34 biosynthetic pathway. In eukaryotes, the biosynthetic pathways of Xm^5^U34 have not been completely clarified: Trm9 and the so-called “Elongator” complex are known to be involved (Huang et al., 2005; Chen et al., 2011; Leihne et al., 2011). Furthermore, although we determined recently that tRNA^Leu^ from *Thermoplasma acidophilum*, a thermo-acidophilic archaeon, has 5-carbamoylmethyluridine at position 34 (ncm^5^U34) (Tomikawa et al., 2013), the biosynthetic pathway in archaea is unknown.

As studies on eukaryotic enzymes have progressed, the number of complex enzymes identified has increased. For example, mammalian enzymes often have additional domains, regulatory subunits and/or paralogs. For information on the identification and prediction of human tRNA methyltransferases, see this review (Towns and Begley, 2012).

## Transfer RNA recognition by tRNA methyltransferases

Transfer RNA methyltransferases strictly modify a specific nucleoside at a specific position in a tRNA. Within the field of nucleic acid-related enzymes, a common question is “How does the enzyme recognize a specific substrate and act at a specific position?” Consequently, the substrate specificities of tRNA methyltransferases have been studied by measuring activities in crude cell extracts, microinjecting labeled tRNA, biochemical studies with purified enzymes, crystallographic studies, and analyses of tRNA from disruptant strains.

In general, tRNA methyltransferases recognize the local structure around the target site in the tRNA, including tertiary structural elements such as stem-loop structure(s). TrmA from *E. coli* recognizes U54 in the ribose-phosphate backbone of the T-arm (Gu and Santi, 1991; Alian et al., 2008). *Aquifex aeolicus* TrmB requires the five nucleotides AGG^*^UC sandwiched between two stem-loop structures (the asterisk corresponds to the methylation site, G46) (Okamoto et al., 2004). TrmFO recognizes the G53-C61 base pair and U54U55C56 sequence in the T-arm (Yamagami et al., 2012). TrmD recognizes the purine36G37 sequence in the anticodon-arm-like microhelix (Brulé et al., 2004; Takeda et al., 2006). In some cases, tertiary interactions are required. For example, crystallographic studies of the complex between Trm5 and tRNA revealed that the enzyme requires interaction between the D- and T-loop of the tRNA (Goto-Ito et al., 2009), which is consistent with the results of biochemical studies with the purified enzyme (Christian et al., 2004; Christian and Hou, 2007).

The target site for methylation is often embedded in the L-shaped tRNA structure. Consequently, in many (or almost all) cases, recognition of tRNA by tRNA methyltransferases seems to involve multiple steps (initial binding and induced fit processes). Although it is very difficult to prepare intermediate complexes, we recently analyzed the initial binding and changes in structure of TrmH by stopped-flow presteady-state kinetic analysis (Ochi et al., 2010, 2013). TrmH binds to tRNA within 10 ms in the initial binding process, in which substrate and non-substrate (methylated) tRNAs are not distinguished. Methylated tRNA is excluded from the complex subsequently due to steric hindrance between the methyl groups in the tRNA and AdoMet before the induced-fit process occurs. The advantage of this mechanism is that methylated tRNA does not severely inhibit the methyl-transfer reaction as a competitive inhibitor. Subsequently, in the induced-fit process, which takes more than 50 ms, G18 is recognized and ribose introduced into the catalytic pocket. During the induced-fit process, movement of Trp126 in motif 2 is observed (Ochi et al., 2013 and Figure 4C).

Several tRNA methyltransferases act on multiple sites in tRNA. For example, archaeal TrmI acts on both A57 and A58 (Roovers et al., 2004; Guelorget et al., 2010). Similarly, *Aquifex aeolicus* Trm1 acts on both G26 and G27 (Awai et al., 2009). On the basis of biochemical studies, we determined that this eubacterial Trm1 recognizes the methylation sites (G26 and G27) from the T-arm (Awai et al., 2009, 2011) whereas archaeal Trm1 recognizes G26 from the D-stem and variable region (Constantinesco et al., 1999b). These Trm1 proteins share high sequence homology (Awai et al., 2009); however, comparison of the crystal structures revealed that the distribution of positive charges on the enzyme surface differs between archaeal (Ihsanawati et al., 2008) and eubacterial (Awai et al., 2011) Trm1. Thus, these studies show how difficult it is to predict target sites on the basis of amino acid sequences. Furthermore, in some cases, other subunits regulate the site specificity. For example, the methylation site recognized by Trm7 is determined by its partner subunit (Guy et al., 2012) and the site specificity of archaeal Trm4 changes in the presence of archease (Auxilien et al., 2007). Moreover, the m^5^C modifications in eukaryotic tRNA are regulated by the presence of an intron in the precursor tRNA (Motorin and Grosjean, 1999; Brzezicha et al., 2006; Auxilien et al., 2012). In addition, some 2′-*O*-methylated nucleosides in archaeal tRNA are introduced by the aFib, Nop5p and L7Ae complex with the BoxC/D guide RNA system (Clouet-d'Orval et al., 2001, 2005; Bortolin et al., 2003; Singh et al., 2004; Renalier et al., 2005; Ye et al., 2009; Joardar et al., 2011; Lin et al., 2011). In some cases, an intron in the precursor tRNA acts as the guide RNA (Clouet-d'Orval et al., 2001, 2005; Bortolin et al., 2003; Singh et al., 2004). This system is useful in minimizing the size of the genome. In the future, it is possible that considerable numbers of 2′-*O*-methylated modifications in archaeal tRNA might be identified as products of this system.

## Regulation of the degradation and localization of tRNA by methylated nucleosides

As shown in Table 1, modifications of the anticodon loop (positions 32–38) are involved directly in protein synthesis whereas other modifications affect the structure of the tRNA. Consequently, for a long time, it was thought that modifications outside the anticodon loop acted to stabilize tRNA structure and regulate the half-life of tRNAs. Indeed, we observed in the thermophilic eubacterium *Thermus thermophiles* that hypomodification at multiple sites in tRNA owing to disruption of one of the modification enzymes promotes the degradation of tRNA^Phe^ and tRNA^Lys^ at high temperatures (Tomikawa et al., 2010).

In the case of eukaryotes, tRNA methylations work coordinately as stabilizing factors and markers of maturation, and the degree of modification changes in response to various stresses. Hypomodified tRNAs are degraded aggressively. For example, in the *Saccharomyces cerevisiae trm4* (synthesizes m^5^C at multiple sites) and *trm8* (produces m^7^G46) double knock-out strain, the half-life of tRNA^Val^ is shortened and the strain shows a growth defect (Alexandrov et al., 2006). Therefore, tRNA modifications stabilize tRNA structure coordinately and systems to degrade hypomodified tRNAs exist in eukaryotic cells (Alexandrov et al., 2006; Chernyakov et al., 2008; Phizicky and Hopper, 2010; D'Silva et al., 2011; Dewe et al., 2012). Furthermore, in *S. cerevisiae*, the m^1^A58 modification by the Trm6–Trm61 complex regulates both the degradation of initiator tRNA^Met^ and its transport from the nucleus to the cytoplasm (Anderson et al., 1998, 2000; Kadaba et al., 2004). The m^1^A58 modification functions a marker of maturation and absence of modification leads to degradation of initiator tRNA^Met^ during transport. Thus, m^1^A58 is part of the RNA quality control system. Moreover, in the case of *S. cerevisiae*, splicing is performed in the cytoplasm (Takano et al., 2005) and precursor tRNAs are matured during repeated-transports between the nucleus and cytoplasm (Ohira and Suzuki, 2011). Therefore, some tRNA modifications might act as the markers of maturation at halfway checkpoints. In *Leishmania tarentolae*, a proportion of tRNA^Glu^ and tRNA^Gln^ is transported from the cytoplasm to the mitochondria (Kaneko et al., 2003). In the cytoplasmic tRNA, U34 is modified to mcm^5^s^2^U34, whereas in the mitochondrial tRNA it is modified to mcm^5^Um34. These results suggest that the s^2^U34 modification may suppress transport from the cytoplasm to mitochondria. Given that both the s^2^U and Um modifications shift the equilibrium of ribose puckering to the C3′-endo form (Kawai et al., 1992), these modifications have a nearly equivalent stabilizing effect on the codon-anticodon interaction. The 5-methylcarboxymethyl (mcm) group restricts wobble base pairing (Takai and Yokoyama, 2003). Taken together, these findings suggest that a substantial number of methylated nucleosides contribute to RNA quality control systems and/or the regulation of tRNA localization, even though they were considered previously to have simply a structural role.

## Adaptation of protein synthesis to environmental change through a network between modified nucleosides and tRNA modification enzymes

### tRNA modifications in *T. thermophilus*

**Figure 5:**
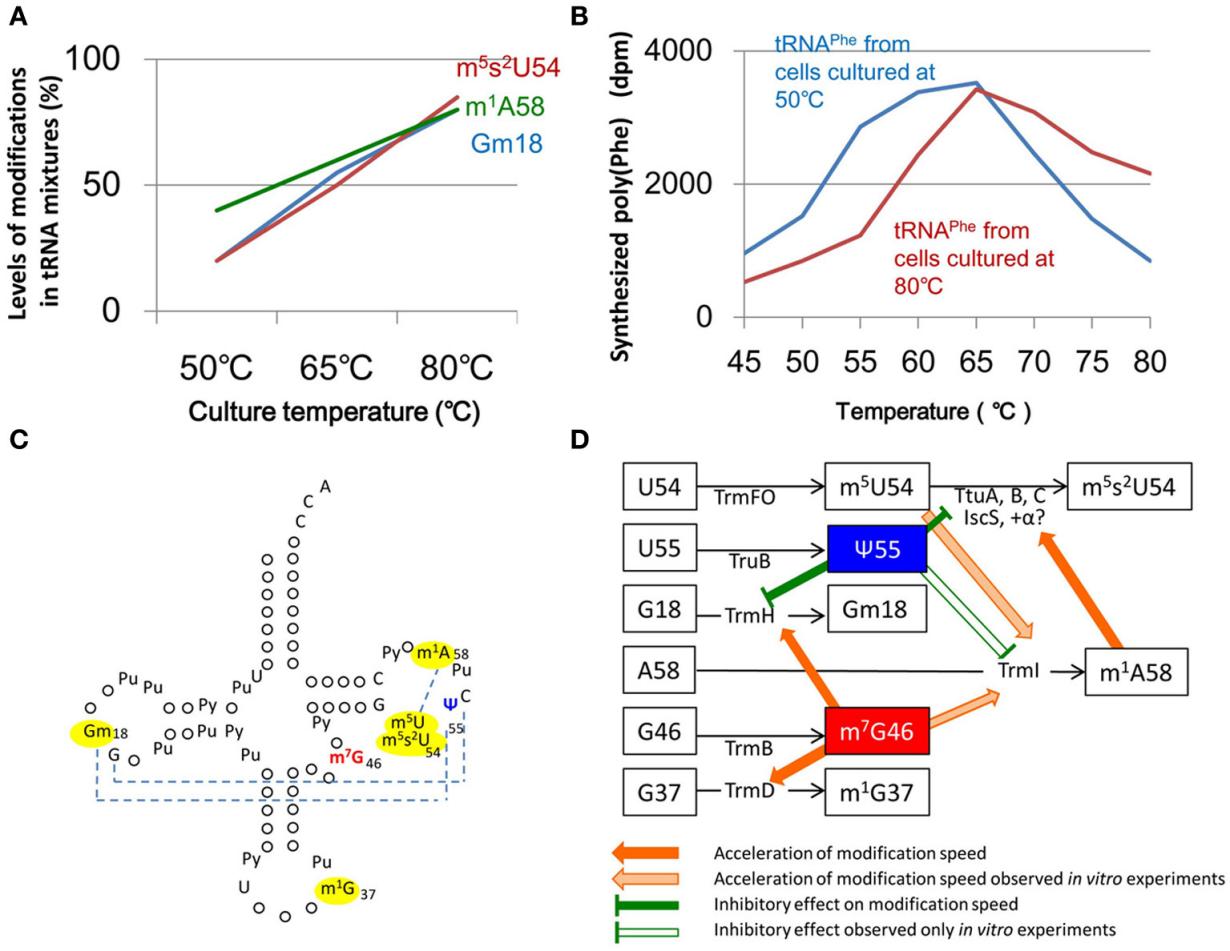
**Network between modified nucleotides and tRNA modification enzymes observed in *T. thermophilus*. (A)** The proportion of Gm18, m^5^s^2^U54, and m^1^A58 in tRNA (contents in tRNA fraction) increases with increasing culture temperature. **(B)** Transfer RNA^Phe^ from cells cultured at 80°C can efficiently synthesize Poly(U) at high temperatures. In contrast, at low temperatures, tRNA^Phe^ from cells cultured at 50°C can work more efficiently than tRNA from cells cultured at 80°C. **(C)** Modifications of tRNA in *T. thermophilus* are depicted on the clover-leaf structure. Dotted lines show the tertiary base pairs. The levels of the m^7^G46 and ψ55 modifications are nearly 100% at a wide range of temperatures. The levels of modifications marked by yellow are regulated by the m^7^G46 and ψ55 modifications. **(D)** At temperatures greater than 65°C, the presence of m^7^G46 increases the rates of modification of Gm18 by TrmH, m^1^A58 by TrmI and m^1^G37 by TrmD. The acceleration of m^1^A58 formation by TrmI in the presence of m^7^G46 and m^5^U54 has been confirmed only by *in vitro* experiments. The m^1^A58 modification accelerates the thio-transfer reaction by the sulfur atom exchange complex that is required for the formation of m^5^s^2^U54. Therefore, at high temperatures, m^7^G46, m^5^U54, and m^1^A58 coordinately promote the formation of m^5^s^2^U54 and increases tRNA stability. In contrast, at low temperatures below 65°C, the ψ55 modification increases rigidity within the local structure of the tRNA as described in the main text. This network provides a mechanism by which extreme thermophilic eubacteria adapt to temperature changes. The network regulates the order of modifications in tRNA. This figure summarizes the experimental data in these publications Yokoyama et al. (1987), Shigi et al. (2006), Tomikawa et al. (2010), Ishida et al. (2011), Yamagami et al. (2012).

*Thermus thermophilus* provides an example of a living organism that utilizes changes in the structural rigidity (flexibility) of tRNA through multiple nucleoside modifications to adapt protein synthesis to environmental changes. *Thermus thermophilus* is an extreme thermophilic eubacterium found in hot springs and can grow at a wide range of temperatures (50~83°C). Under natural conditions, the temperature of hot springs can be changed dramatically by several factors, for instance the overflow of hot spring water, snow falling, and the influx of river water. *Thermus thermophilus* can synthesize proteins in response to these temperature changes. Three distinct modifications (Gm18, m^5^s^2^U54, and m^1^A58) are found in *T. thermophilus* tRNA and the combination of these modifications increases the melting temperature of tRNA by near 10°C as compared with that of the unmodified transcript (Watanabe et al., 1976; Horie et al., 1985; Shigi et al., 2006; Tomikawa et al., 2010). Although these modifications are very important as structural factors in tRNA, they do not have an effect on translational fidelity below 65°C and the level of modification is very low in tRNA from cells cultured at 50°C (Figure 5A). This change in the extent of modification reflects the adaptation of protein synthesis to temperature change (Yokoyama et al., 1987). Transfer RNA^Phe^ from cells cultured at 80°C efficiently synthesizes poly(U) at high temperatures (above 65°C). In contrast, tRNA^Phe^ from cells cultured at 50°C, in which the levels of the three modifications are low, works efficiently at low temperatures (Figure 5B). Thus, the levels of three modified nucleosides, Gm18, m^5^s^2^U54, and m^1^A58, in tRNA control the elongation of translation *via* the flexibility of the tRNA. These findings were reported in 1987 (Yokoyama et al., 1987). However, at the beginning of the twenty-first century, the mechanisms of regulation of these modifications remained unknown.

### Switching of network between modified nucleosides and tRNA modification enzymes

Initially, we assumed that transcriptional and/or translational regulation of the tRNA modification enzymes was involved in the regulation of the three modifications. However, unexpectedly, we have observed that the phenomenon can be simply explained by the RNA recognition mechanisms of the tRNA modification enzymes (Shigi et al., 2002; Tomikawa et al., 2010; Ishida et al., 2011; Yamagami et al., 2012). Several common modifications (for example, m^7^G46 and ψ55) are found in *T. thermophilus* tRNA in addition to Gm18, m^5^s^2^U54, and m^1^A58. When the genes for the modification enzymes for m^7^G46 and ψ55 (*trmB* and *truB*, respectively) were disrupted individually, the levels of Gm18, m^5^s^2^U54 and m^1^A58 in tRNA were changed dramatically (Tomikawa et al., 2010; Ishida et al., 2011). Thus, modified nucleosides and tRNA modification enzymes form a network, and this network regulates the extent of modifications on the basis of temperature (Figures 5C,D).

At high temperatures (above 65°C), m^7^G46 functions as a marker of precursor tRNA and increases the reaction rates of other modification enzymes. In contrast, at low temperatures, ψ55 confers local structural rigidity and slows down the rate of formation of other modifications around ψ55 (that is, Gm18, m^5^s^2^U54, and m^1^A58). This inhibitory effect weakens as the temperature increases and is not observed above 65°C. Thus, the m^7^G46 and ψ55 modifications work as an accelerator and a brake in the network, respectively. The advantage of this mechanism is that the network does not include any transcriptional or translational regulatory steps: protein synthesis is not necessary. Thus, the response of the network to environmental changes is very rapid. This is a typical strategy in eubacteria, where genome size is limited.

Similar networks between modified nucleosides and tRNA modification enzymes have also been reported in mesophiles. For example, ms^2^i^6^A37 modification in *E. coli* tRNA is required for 2′-*O*-methylation by TrmL (Benítez-Páez et al., 2010), and the Cm32 and Gm34 modifications in *S. cerevisiae* tRNA^Phe^ are required for the formation of yW37 from m^1^G37 (Guy et al., 2012). However, the network in *T. thermophilus* is distinct because the modifications are almost all in the three-dimensional core of the tRNA and the network responds to environmental changes.

## Genetic disease and tRNA methylation

Modifications of tRNA regulate protein synthesis. Consequently, if a disruption of tRNA modification is not lethal, it can directly cause a genetic disease. In fact, there are several reports concerning the relationship between genetic disease and tRNA modification (Yasukawa et al., 2001; Suzuki et al., 2002, 2011b; Freude et al., 2004; Kirino et al., 2005; Umeda et al., 2005; Wei et al., 2011; Towns and Begley, 2012; Igoillo-Esteve et al., 2013). In particular, the number of reports of a link between diabetes and tRNA modification are increasing, which suggests that an increase in the frequency of translation errors has an effect on energy metabolism. The severe disruption of energy metabolism often damages muscle and neuronal cells, which consume large amounts of energy. This perspective enables mitochondrial diseases that are caused by a problem with mitochondrial tRNA modification to be understood (Yasukawa et al., 2001; Suzuki et al., 2002, 2011b; Kirino et al., 2005; Umeda et al., 2005). Furthermore, several tRNA methyltransferases are fused to DNA repair enzymes, which means that these enzymes are related directly to DNA repair and carcinogenesis (Choudhury et al., 2007a,b; Shimada et al., 2009). Moreover, abnormal tRNA modifications have been also reported in cancers (Kuchino and Borek, 1978; Kuchino et al., 1981; Shindo-Okada et al., 1981). These might be caused by the rearrangement of chromosomes in cancer cells.

## Infection, immunity, and tRNA methylations—tRNA therapy

Among the tRNA modification enzymes, tRNA guanine transglycosidase (Tgt), which is required for the production of Q34, and tRNA ψ55 synthase (TruB), which generates ψ55, are essential factors for infection by *Shigella flexneri* (Durand et al., 1994) and *Pseudomonas aeruginosa* (Saga et al., 1997), respectively. Similarly, we also found that tRNA (m^7^G46) methyltransferase is essential for infection by *Colletotrichum lagenarium*, an infectious fungus (Takano et al., 2006). Furthermore, tRNAs that contain mcm^5^U modifications are the target of *Kluyveromyces lactis* gamma-toxin (Lu et al., 2005) and *Pichia acaciae* killer toxin (Klassen et al., 2008). Moreover, given that retroviruses utilize host tRNA as the primer for reverse transcription, tRNA methylation and methyltransferases are involved in both reverse transcription and the packaging of virus particles. For example, human immunodeficiency virus (HIV; AIDS virus) utilizes the m^1^A58 modification in tRNA^Lys^3 as the terminator of reverse transcription (see reviews, Marquet, 1998; Saadatmand and Kleiman, 2012; Sleiman et al., 2012). Consequently, the regulation of tRNA modification and modification enzymes might be a powerful tool to control infectious organisms.

**Figure 6:**
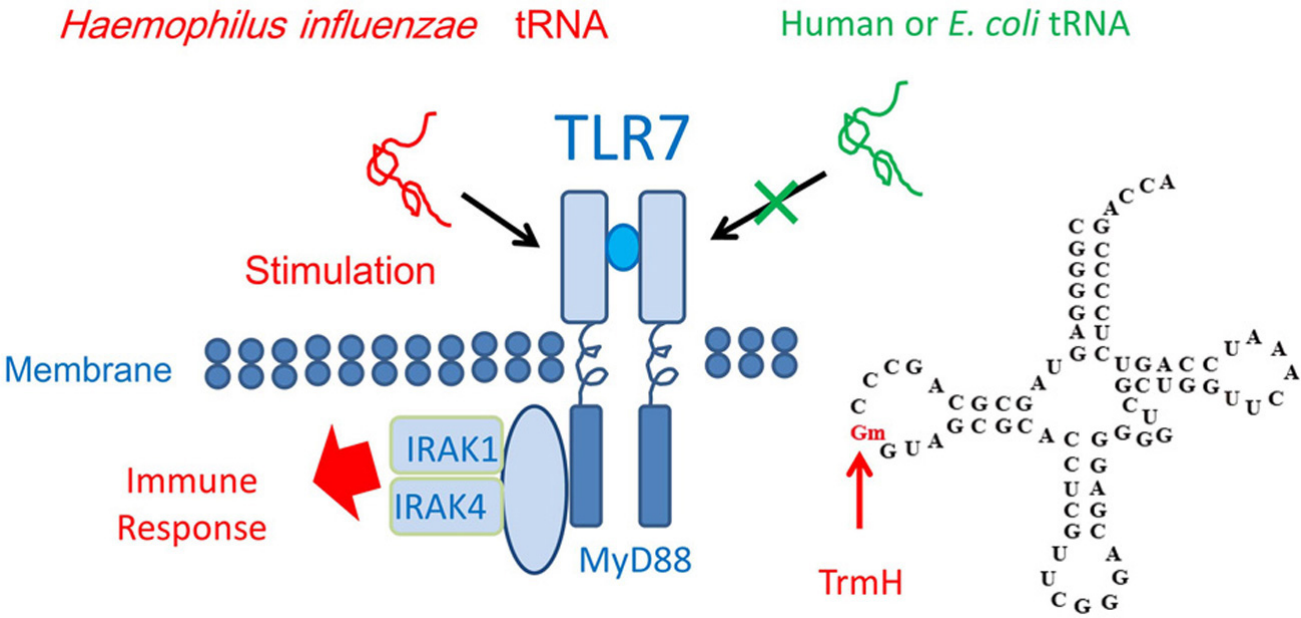
**Immune response and tRNA methylation**. Transfer RNA from *H. influenzae*, a respiratory infectious bacterium, induces the dimer formation by Toll like receptor-7 (TLR7) and then the immune response is stimulated *via* binding of the proteins, MyD88, IRAK1, and IRAK4. However, human and *E. coli* tRNAs do not stimulate TLR7 because these tRNA contains the Gm18 modification. The *E. coli trmH* gene disruptant strain does not show any obvious phenotype under laboratory culture conditions (Persson et al., 1997). The Gm18 modification and TrmH are required for survival of *E. coli* in the animal gut.

When an exogenous single-stranded RNA such as *Haemophilus influenzae* tRNA is present in humans, Toll-like receptor 7 (TLR7) forms a dimer structure and then activates the immune response systems (Figure 6). However, endogenous or *E. coli* tRNA does not stimulate TLR7. The mechanism of differentiation was clarified recently by two groups, who found that the Gm18 modification in *E. coli* tRNA suppresses immunostimulation *via* TLR7 (Gehrig et al., 2012; Jöckel et al., 2012). Thus, enterobacteria exploit the Gm18 modification in tRNA to avoid the host immune system. Furthermore, given that Gm18-modified tRNA acts as an antagonist of TLR7 (Jöckel et al., 2012), Gm18-modified tRNA might be an effective anti-inflammatory drug.

## Evolution of modifications in tRNA

Finally, it is worthwhile discussing the evolution of modifications in tRNA. During the early period of chemical evolution (see reviews Cermakian and Cedergren, 1998; Joyce and Orgel, 2006), inosine could be used as a basic component of RNA, because it can be synthesized from adenosine non-enzymatically. Inosine seems to have been excluded after the appearance of genes because it changes the genetic information during the replication process. Simple methylated nucleosides such as m^1^G became essential when the reading frame of protein synthesis was separated into three-nucleotide units (Björk et al., 1989, 2001). Thus, several methylated nucleosides seem to have appeared during the chemical evolution period (Cermakian and Cedergren, 1998). After the appearance of the reading frame, the importance of the availability of methyl groups increased and it seems that the methionine codon was selected as the translation initiation codon.

It appears that complicated enzymes were not formed during the period of chemical evolution (Joyce and Orgel, 2006). The early enzymes might have been oligopeptides and might have included metals as the catalytic center, as is the case for deaminases (Carter, 1998; Schaub and Keller, 2002). It is possible that the codons were not fixed strictly as is observed in the universal code (Jukes, 1973; Cedergren et al., 1986; Osawa et al., 1992). However, it is likely that the most basic catalytic core of tRNA methyltransferases was established when cell-like organisms began to exchange their components and genes because the basic structure of tRNA methyltransferases is shared by all living organisms found today (Figure 4 and Table 2). The structures of methyltransferases (Schubert et al., 2003) suggest that RNA methyltransferases, which were required for protein synthesis, evolved to yield DNA and protein methyltransferases many times during the evolution of life. The mechanisms to generate the complicated modified nucleotides that regulate the wobble base pair seem to have arisen after the origination of living organisms because they show considerable diversity and involve multistep reactions (Table 1).

The temperature of primordial Earth was higher than that of the Earth at present. Consequently, several nucleoside modifications in tRNA and rRNA would be necessary to stabilize the structure of the RNA (Motorin and Helm, 2010). However, it is likely that the network between modified nucleosides and tRNA modification enzymes that is observed in extreme thermophiles (Figure 5D and section Adaptation of Protein Synthesis to Environmental Change Through a Network Between Modified Nucleosides and tRNA Modification Enzymes) was established after the cooling of the Earth because it responds to low temperatures (Ishida et al., 2011). Obviously, the functions of modified nucleotides with respect to the RNA quality control system and regulation of cellular localization were acquired after the appearance of eukaryotes (see section Regulation of the Degradation and Localization of tRNA by Methylated Nucleosides).

Transfer RNA modifications are still evolving. The most powerful driving force is the existence of infectious organisms (see section Infection, Immunity, and tRNA Methylations—tRNA Therapy). Hosts need to distinguish endogenous RNA from exogenous RNA to prevent infection and infectious organisms need to avoid the host defense system to survive. Consequently, tRNA modifications and modification enzymes are still subject to evolution even today.

### Conflict of interest statement

The author declares that the research was conducted in the absence of any commercial or financial relationships that could be construed as a potential conflict of interest.
